# Association of fermented food intake with the prevalence of depressive symptoms and suicidal ideation in men and women stratified by age: the Korea National Health and Nutrition Examination Survey 2014–2022

**DOI:** 10.3389/fnut.2026.1707954

**Published:** 2026-01-30

**Authors:** Taehoon Kim, Yongsoon Park

**Affiliations:** Department of Food and Nutrition, Hanyang University, Seoul, Republic of Korea

**Keywords:** age-related interaction, depression, fermented food, Korean National Health and Nutritional Examination Survey, sex-specific differences, suicidal ideation

## Abstract

**Introduction:**

Associations between fermented foods and the risk of depression and suicidal ideation have been suggested; however, the effects of fermented food types and sex-specific differences remain unknown. The present study investigated the hypothesis that the association of fermented food intake and types with the prevalence of depressive symptoms and suicidal ideation differs between men and women. Age-related interactions were evaluated for the association.

**Methods:**

Using data from the Korea National Health and Nutrition Examination Survey 2014–2022, 8,747 men and 12,449 women aged 19–79 years were analyzed. Depressive symptoms were defined as a Patient Health Questionnaire-9 (PHQ-9) score ≥10 and suicidal ideation as a score ≥1 on the ninth question of PHQ-9.

**Results:**

The intake of fermented foods, soy products, and vegetables was inversely associated with the prevalence of depressive symptoms and suicidal ideation in the total population. The prevalence of depressive symptoms was inversely associated with the intake of fermented soy products and vegetables in women and with the intake of fermented dairy products in men. Additionally, the prevalence of suicidal ideation was inversely associated with fermented soy product intake in women. A significant age-related interaction was observed between fermented soy products and prevalence of depressive symptoms in women.

**Conclusion:**

The intake of fermented foods was inversely associated with the prevalence of depressive symptoms and suicidal ideation with sex-specific differences, suggesting that fermented foods could be beneficial for preventing depressive symptoms and suicidal ideation in Korean adults.

## 1 Introduction

Depression is a mood disorder characterized by persistent sadness and/or an inability to experience pleasure and impaired daily functioning (1). According to the World Health Organization, the prevalence of depression is 4.4% globally and depression is a major contributor to suicide (2). Furthermore, the prevalence of depression is more than twice as high in women than that in men, with higher rates in younger adults than that in older adults in high-income countries (3). Previous meta-analysis showed that patients with depression had higher levels of blood inflammatory cytokines such as interleukin-1β (IL-1β) and tumor necrosis factor-*α* (TNF-α) compared with healthy controls (4). Our previous studies demonstrated that supplementation of probiotics and postbiotics modulated gut microbiome, decreased the secretion of inflammatory cytokines such as IL-1β and TNF-*α*, and improved depressive behaviors through serotonergic and dopaminergic pathways in depressed rats induced by chronic mild stress (5, 6). A systematic review of observational studies reported that pro-inflammatory gut bacteria were higher, while butyrate-producing gut bacteria were lower in patients with depression than those in healthy controls, suggesting a link between depression and the gut microbiota (7). Zhang et al. (8) showed that the consumption of fermented milk increased the number of beneficial gut bacteria while reducing mental illness-associated gut bacteria compared with that using placebo beverages in Chinese patients diagnosed with both depression and constipation.

A meta-analysis of randomized controlled trials demonstrated that supplementation with probiotics, prebiotics, or synbiotics improved depressive symptoms in patients with mild to moderate depression in Asian and Western population (9). Epidemiological studies have also shown that the estimated intake of live microbes, categorized into three levels according to the expected microbial content in each food, was inversely associated with the risk of depression among American adults according to the National Health and Nutrition Examination Survey (NHANES) (10–12).

The risk of depression is inversely associated with the intake of fermented foods in Korean (13), American (14), and Polish adults (15). In addition, fermented soy product intake is inversely associated with the risk of depression in pregnant Japanese women (16). The intake of fermented dairy products was also inversely associated with the risk of depression in middle-aged and older Finnish men (17). In contrast, yogurt or cheese intake was not significantly associated with the risk of depression in women in Japan (18), Saudi Arabia (19), and Spain (20), suggesting sex-specific differences in fermented foods. Fermented foods are rich in lactic acid bacteria, especially *Lactobacillus* species, known to exert anti-depressive effects (21).

Furthermore, previous studies have reported that the abundance of gut bacteria involved in inflammation, serotonin metabolism, and adrenal steroid hormones is higher in participants with suicidal ideation or completion than in those without (22–24). The intake of dietary live microbes, estimated based on the expected microbial content of foods, was also inversely associated with the risk of suicidal ideation among American adults in the NHANES (10).

To the best of our knowledge, there have been few studies on the association between the type of fermented food consumed and the risk of depression and suicidal ideation, with sex-specific differences. Thus, the present study aimed to investigate whether the beneficial association of fermented food intake and its types with the prevalence of depressive symptoms and suicidal ideation differs between men and women. In addition, age-related interactions in the association between fermented food intake and prevalence of depressive symptoms and suicidal ideation were determined.

## 2 Materials and methods

### 2.1 Study population

The Korean National Health and Nutritional Examination Survey (KNHANES) uses a complex, stratified, multistage, and probability-cluster sampling design to obtain a nationally representative sample consisting of three components: a health interview, health examination, and nutrition survey (25). Data from KNHANES 2014, 2016, 2018, 2020, and 2022 was used, since the Patient Health Questionnaire-9 (PHQ-9) was administered. The KNHANES protocols were approved by the Institutional Review Board of the Korea Centers for Disease Control and Prevention and informed consent was obtained from all participants. This study was approved by the Institutional Review Board of Hanyang University (HYUIRB-202508-026).

**Figure 1:**
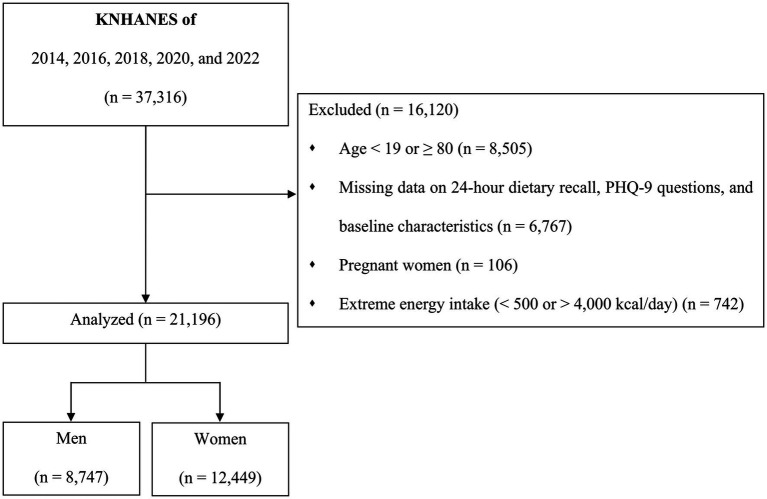
Flowchart for the inclusion of study participants. KNHANES, Korean National Health and Nutrition Examination Survey; PHQ-9, Patient Health Questionnaire-9.

Among the total of 37,316 participants, 16,120 were excluded for the following reasons: aged <19 years or ≥80 years (*n* = 8,505); no data on 24-h dietary recall (*n* = 3,952) and PHQ-9 questions (*n* = 2,557); pregnant women (*n* = 106); extreme energy intake of <500 kcal/day or >4,000 kcal/day (*n* = 742); and missing baseline characteristics such as BMI (*n* = 99), education (*n* = 9), household income (*n* = 39), smoking status (*n* = 64), and physical activity (*n* = 47). The final analysis included 21,196 participants (8,747 men and 12,449 women) (Figure 1). In KNHANES, PHQ-9 was not performed in Koreans aged <19 years, and thus we excluded those aged <19 years in the present study. In addition, we excluded participants aged ≥80 years because the prevalence of cognitive impairment and dementia was higher among older adults aged ≥80 years (26), which could reduce the reliability of self-reported questionnaires.

### 2.2 Assessment of depressive symptoms and suicidal ideation

Moderate depressive symptoms were defined as a PHQ-9 score of 10 or higher, which corresponds to a threshold that demonstrates 88% sensitivity and 88% specificity for detecting major depressive disorders (27). The Korean version of the PHQ-9 consists of nine items from the Diagnostic and Statistical Manual of Mental Disorders, 4th Edition (DSM-IV), each rated on a scale from not at all (0) to several days (1), more than half of the days (2), and nearly every day (3) during the previous 2 weeks, resulting in a total score ranging from 0 to 27.

Suicidal ideation was measured using ninth item of the PHQ-9, which was “in the past 2 weeks, have you ever had the thoughts that you would be better off dead, or of hurting yourself in some way?” (28). If the score of the ninth item was 1 or greater, the participant was defined as having suicidal ideation.

### 2.3 Assessment of dietary intake

Dietary intake was assessed through 24-h recall conducted by trained dietitians who asked participants to report all foods and beverages consumed on the previous day. Fermented foods were identified and quantified using standardized food codes, and subsequently categorized into four groups: fermented soy products, vegetables, dairy products, and seafood. Fermented soy products include *gochujang* (fermented red pepper paste), *doenjang* (soybean paste fermented for 3–6 months), *ganjang* (fermented soy sauce), and *cheonggukjang* (soybean paste fermented for 2–3 days) (29). Fermented vegetables include *kimchi* (fermented napa cabbage, radish, young radish, cucumber, scallion, and other leafy greens) and *jangajji* (other fermented vegetables). Fermented dairy products include yogurt and cheese, and fermented seafood includes *jeotgal* (fermented fish, shellfish, and fish roe).

### 2.4 Confounding variables

Data on age, sex, education, household income, smoking status, alcohol consumption, physical activity, and employment were obtained through interviews. Age was categorized into three groups: 19–44 years (young adults), 45–64 years (middle-aged adults), and ≥65 years (older adults). Physical activity was assessed using the Global Physical Activity Questionnaire, which included the type, intensity, and duration of physical activity performed. Based on total metabolic equivalent of task (MET)-min per week, participants were classified as inactive (0 MET-min/week), moderate (1–599 MET-min/week), or vigorous (≥600 MET-min/week). Body mass index (BMI) was calculated using the measured height and weight, which were recorded to the nearest 0.1 cm and 0.1 kg, respectively, with the participants wearing light clothing and barefoot.

### 2.5 Statistical analysis

All analyses were performed using sampling weights, stratification, and clustering to account for the complex survey design of the KNHANES and to obtain nationally representative estimates (25). Baseline characteristics are presented as weighted mean (standard error of the mean) for continuous variables and as number (weighted percentage) for categorical variables. Differences between participants with and without depressive symptoms were compared using independent *t*-tests for continuous variables and chi-square tests for categorical variables.

In the multivariate regression models, covariates with a *p*-value < 0.20, such as age, sex, BMI, education, household income, smoking status, physical activity, and unemployment, were selected as potential covariates (30). Differences in fermented food intake were assessed using analysis of covariance between participants with and without depressive symptoms after adjusting for potential covariates. Associations between fermented food intake, depressive symptoms, and suicidal ideation were evaluated using multivariable logistic regression analyses after adjusting for potential covariates, with intake as a continuous variable and tertile. In addition, *p*-values for the trends were estimated using the median value of fermented food intake within each tertile. Subgroup analyses and interaction tests for the age group were performed using multivariate logistic regression after adjusting for potential covariates. All statistical analyses were performed using SAS software (version 9.4; SAS Institute, Cary, NC, USA) and a *p*-value < 0.05 was considered statistically significant.

## 3 Results

### 3.1 Baseline characteristics of participants

Participants with depressive symptoms had a higher proportion of those aged 19–44 years, while a lower proportion of those aged 45–65 years as compared with those without depressive symptoms among total population, men, and women (Table 1). Regarding education levels, participants with depressive symptoms had higher proportion of ≥college graduates, while lower proportion of ≤middle school and high school graduates as compared with those without depressive symptoms among total population, men, and women. In addition, participants with depressive symptoms had higher proportion of lowest and lower middle household income, while lower proportion of upper middle and highest household income as compared with those without depressive symptoms among total population, men, and women. As compared with participants without depressive symptoms, those with depressive symptoms had higher proportion of inactive physical activity, while a lower proportion of moderate and vigorous physical activities among total population, men, and women. Participants with depressive symptoms had a higher proportion of smokers and unemployed individuals as compared with those without depressive symptoms in total population, men, and women. Participants with depressive symptoms had a higher BMI in women, a higher proportion of non-drinkers in the total population, and lower energy intake as compared with those without depressive symptoms in the total population and men.

**Table 1:** Baseline characteristics of participants with and without depressive symptoms.

Variables	Total population		*p* value	Men		*p* value	Women		*p* value
	Without depressive symptoms (*n* = 20,072)	With depressive symptoms (*n* = 1,124)		Without depressive symptoms (*n* = 8,429)	With depressive symptoms (*n* = 318)		Without depressive symptoms (*n* = 11,643)	With depressive symptoms (*n* = 806)	
Age, *n* (%)			<0.001			0.001			0.001
19–44 years	7,382 (45.4)	437 (51.7)		3,060 (47.0)	140 (58.7)		4,322 (43.9)	297 (47.8)	
45–64 years	7,891 (39.1)	379 (31.5)		3,154 (37.9)	101 (29.3)		4,737 (40.2)	278 (32.8)	
≥65 years	4,799 (15.5)	308 (16.8)		2,215 (15.0)	77 (12.0)		2,584 (16.0)	231 (19.5)	
BMI (kg/m^2^)	24.0 ± 0.0	24.3 ± 0.2	0.059	24.7 ± 0.1	25.1 ± 0.3	0.254	23.3 ± 0.1	23.9 ± 0.2	0.001
Education, *n* (%)			<0.001			0.049			<0.001
≤Middle school	5,522 (19.7)	459 (28.6)		1,967 (15.8)	103 (19.8)		3,555 (23.5)	356 (33.6)	
High school	6,784 (36.9)	367 (38.0)		2,965 (37.9)	118 (41.4)		3,819 (35.9)	249 (36.1)	
≥College	7,766 (43.5)	298 (33.4)		3,497 (46.3)	97 (38.7)		4,269 (40.7)	201 (30.4)	
Household income, *n* (%)			<0.001			<0.001			<0.001
Lowest	3,223 (12.9)	398 (29.2)		1,269 (11.8)	120 (31.0)		1,954 (13.9)	278 (28.2)	
Lower middle	4,962 (23.3)	288 (25.3)		2,032 (22.5)	83 (25.1)		2,930 (24.1)	205 (25.4)	
Upper middle	5,748 (30.1)	265 (25.8)		2,467 (30.8)	60 (21.8)		3,281 (29.4)	205 (28.0)	
Highest	6,139 (33.7)	173 (19.7)		2,661 (34.9)	55 (22.0)		3,478 (32.5)	118 (18.4)	
Smoker, *n* (%)	3,206 (19.2)	280 (29.6)	<0.001	2,706 (33.8)	159 (53.2)	<0.001	500 (4.8)	121 (16.5)	<0.001
Nondrinker, *n* (%)	9,478 (42.9)	604 (47.9)	0.006	2,658 (30.2)	108 (32.4)	0.466	6,820 (55.3)	496 (56.5)	0.576
Physical activity, *n* (%)			<0.001			0.004			0.014
Inactive	5,927 (27.0)	419 (33.6)		2,517 (27.4)	130 (36.1)		3,410 (26.6)	289 (32.2)	
Moderate	4,806 (23.2)	236 (20.3)		1,749 (20.0)	47 (14.0)		3,057 (26.3)	189 (23.9)	
Vigorous	9,339 (49.8)	469 (46.0)		4,163 (52.6)	141 (49.9)		5,176 (47.1)	328 (43.9)	
Unemployment, *n* (%)	7,603 (34.6)	661 (55.6)	<0.001	2,256 (24.0)	163 (46.7)	<0.001	5,347 (45.1)	498 (60.5)	<0.001
Energy intake (kcal/day)	1,921 ± 7	1,781 ± 29	<0.001	2,186 ± 10	2,068 ± 53	0.028	1,660 ± 7	1,621 ± 29	0.201

BMI, body mass index; data are presented as weighted mean ± standard error of the mean or number of participants (weighted percentage distribution) as appropriate. *p*-values were analyzed using independent *t*-tests for continuous variables and chi-squared tests for categorical variables.

### 3.2 Associations of fermented food intake with depressive symptoms and suicidal ideation

Compared with participants without depressive symptoms, those with depressive symptoms consumed significantly less fermented food and soy products by the total population and women, fewer fermented vegetables by women, and fewer fermented dairy products by men (Supplementary Table 1). The prevalence of depressive symptoms was inversely associated with the intake of fermented food and soy products in the total population and women, and with the intake of fermented vegetables in women as a continuous and non-continuous variable (Table 2). Additionally, the prevalence of depressive symptoms was inversely associated with the intake of fermented food in men and fermented vegetables in the total population as a non-continuous variable, and fermented dairy products in men as a continuous variable. Fermented dairy products and fermented seafood were not divided into tertiles because 70–80% of the participants did not consume these foods.

**Table 2:** Associations of fermented food intake with prevalence of depressive symptoms in study population.

Variables	Tertiles of dietary intake			*p* for trend^1^	Dietary intake continuous^2^	
	T1	T2	T3		OR (95% CI)	*p*
Total population						
Fermented food (g/day)	≤65.2	65.2< to ≤156.4	>156.4			
With/without depressive symptoms, *n*	488/6,577	329/6,735	307/6,760			
OR (95% CI)	1.0 (ref)	0.72 (0.60–0.85)	0.74 (0.62–0.90)	0.004	0.991 (0.984–0.999)	0.018
Fermented soy products (g/day)	≤6.4	6.4< to ≤19.0	>19.0			
With/without depressive symptoms, *n*	474/6,591	341/6,724	309/6,757			
OR (95% CI)	1.0 (ref)	0.79 (0.67–0.94)	0.80 (0.67–0.97)	0.037	0.948 (0.907–0.991)	0.019
Fermented vegetable (g/day)	≤41.6	41.6< to ≤117.7	>117.7			
With/without depressive symptoms, *n*	462/6,602	347/6,719	315/6,751			
OR (95% CI)	1.0 (ref)	0.83 (0.70–0.98)	0.78 (0.64–0.94)	0.011	0.993 (0.985–1.001)	0.103
Fermented dairy products (g/day)					0.990 (0.972–1.008)	0.273
Fermented seafood (g/day)					0.989 (0.867–1.128)	0.868
Men						
Fermented food (g/day)	≤87.2	87.2< to ≤185.8	>185.8			
With/without depressive symptoms, *n*	143/2,773	84/2,830	91/2,826			
OR (95% CI)	1.0 (ref)	0.70 (0.50–0.99)	0.66 (0.48–0.92)	0.016	0.993 (0.982–1.004)	0.201
Fermented soy products (g/day)	≤8.0	8.0< to ≤22.6	>22.6			
With/without depressive symptoms, *n*	138/2,778	94/2,820	86/2,831			
OR (95% CI)	1.0 (ref)	0.69 (0.49–0.96)	0.78 (0.56–1.08)	0.186	0.960 (0.895–1.029)	0.251
Fermented vegetable (g/day)	≤57.1	57.1< to ≤147.1	>147.1			
With/without depressive symptoms, *n*	127/2,788	99/2,816	92/2,825			
OR (95% CI)	1.0 (ref)	0.96 (0.69–1.33)	0.77 (0.55–1.09)	0.132	0.997 (0.986–1.009)	0.666
Fermented dairy products (g/day)					0.956 (0.916–0.998)	0.038
Fermented seafood (g/day)					1.066 (0.937–1.213)	0.332
Women						
Fermented food (g/day)	≤54.5	54.5< to ≤135.5	>135.5			
With/without depressive symptoms, n	328/3,821	243/3,907	235/3,915			
OR (95% CI)	1.0 (ref)	0.69 (0.56–0.85)	0.75 (0.61–0.92)	0.019	0.990 (0.981–0.999)	0.039
Fermented soy products (g/day)	≤5.6	5.6< to ≤16.7	>16.7			
With/without depressive symptoms, *n*	335/3,815	245/3,903	226/3,925			
OR (95% CI)	1.0 (ref)	0.79 (0.65–0.98)	0.75 (0.60–0.93)	0.015	0.940 (0.886–0.997)	0.040
Fermented vegetable (g/day)	≤31.2	31.2< to ≤94.3	>94.3			
With/without depressive symptoms, *n*	328/3,822	236/3,913	242/3,908			
OR (95% CI)	1.0 (ref)	0.74 (0.60–0.91)	0.70 (0.57–0.87)	0.002	0.990 (0.980–0.999)	0.043
Fermented dairy products (g/day)					0.999 (0.980–1.020)	0.957
Fermented seafood (g/day)					0.874 (0.702–1.088)	0.228

OR, odd ratios; CI, confidence intervals; BMI, body mass index.

Logistic regression analysis was adjusted for age, sex, BMI, education, household income, smoking status, physical activity, and unemployment.

1 Estimates of *p* values for linear trends were based on linear scores derived from the medians of the tertiles of fermented food intake among all participants.

2 Each point corresponds to a 10-unit increase in fermented food intake.

The prevalence of suicidal ideation was inversely associated with the intake of fermented foods and vegetables in women as continuous and non-continuous variables (Table 3). Additionally, the prevalence of suicidal ideation was inversely associated with the intake of fermented food and vegetables in the total population and men, and intake of fermented soy products in the total population and women as non-continuous variables.

**Table 3:** Associations of fermented food intake with prevalence of suicidal ideation in study population.

Variables	Tertiles of dietary intake			*p* for trend^1^	Dietary intake continuous^2^	
	T1	T2	T3		OR (95% CI)	*p*
Total population						
Fermented food (g/day)	≤65.2	65.2< to ≤156.4	>156.4			
With/without suicidal ideation, *n*	515/6,550	362/6,702	342/6,725			
OR (95% CI)	1.0 (ref)	0.72 (0.61–0.85)	0.67 (0.56–0.80)	<0.001	0.993 (0.985–1.000)	0.067
Fermented soy products (g/day)	≤6.4	6.4< to ≤19.0	>19.0			
With/without suicidal ideation, *n*	519/6,546	366/6,699	334/6,732			
OR (95% CI)	1.0 (ref)	0.78 (0.67–0.92)	0.75 (0.63–0.89)	0.003	0.964 (0.924–1.005)	0.084
Fermented vegetable (g/day)	≤41.6	41.6< to ≤117.7	>117.7			
With/without suicidal ideation, *n*	481/6,583	380/6,686	358/6,708			
OR (95% CI)	1.0 (ref)	0.86 (0.73–1.03)	0.70 (0.58–0.84)	<0.001	0.994 (0.986–1.002)	0.160
Fermented dairy products (g/day)					0.994 (0.977–1.010)	0.450
Fermented seafood (g/day)					0.921 (0.799–1.063)	0.260
Men						
Fermented food (g/day)	≤87.2	87.2< to ≤185.8	>185.8			
With/without suicidal ideation, *n*	170/2,746	105/2,809	124/2,793			
OR (95% CI)	1.0 (ref)	0.64 (0.48–0.86)	0.72 (0.55–0.96)	0.035	0.996 (0.986–1.007)	0.514
Fermented soy products (g/day)	≤8.0	8.0< to ≤22.6	>22.6			
With/without suicidal ideation, *n*	164/2,752	131/2,783	104/2,813			
OR (95% CI)	1.0 (ref)	0.79 (0.60–1.05)	0.75 (0.55–1.03)	0.098	0.976 (0.916–1.040)	0.452
Fermented vegetable (g/day)	≤57.1	57.1< to ≤147.1	>147.1			
With/without suicidal ideation, *n*	167/2,748	115/2,800	117/2,800			
OR (95% CI)	1.0 (ref)	0.70 (0.52–0.93)	0.64 (0.48–0.85)	0.004	0.998 (0.986–1.010)	0.733
Fermented dairy products (g/day)					0.993 (0.968–1.018)	0.574
Fermented seafood (g/day)					0.881 (0.681–1.138)	0.330
Women						
Fermented food (g/day)	≤54.5	54.5< to ≤135.5	>135.5			
With/without suicidal ideation, *n*	335/3,814	244/3,906	241/3,909			
OR (95% CI)	1.0 (ref)	0.69 (0.56–0.84)	0.67 (0.54–0.83)	0.001	0.989 (0.980–0.999)	0.031
Fermented soy products (g/day)	≤5.6	5.6< to ≤16.7	>16.7			
With/without suicidal ideation, *n*	347/3,803	251/3,897	222/3,929			
OR (95% CI)	1.0 (ref)	0.82 (0.67–1.00)	0.70 (0.57–0.86)	0.001	0.951 (0.900–1.004)	0.071
Fermented vegetable (g/day)	≤31.2	31.2< to ≤94.3	>94.3			
With/without suicidal ideation, *n*	311/3,839	249/3,900	260/3,890			
OR (95% CI)	1.0 (ref)	0.80 (0.65–0.98)	0.74 (0.60–0.91)	0.009	0.990 (0.980–0.999)	0.045
Fermented dairy products (g/day)					0.993 (0.971–1.015)	0.545
Fermented seafood (g/day)					0.991 (0.847–1.161)	0.915

OR, odd ratios; CI, confidence intervals; BMI, body mass index.

Logistic regression analysis was adjusted for age, sex, BMI, education, household income, smoking status, physical activity, and unemployment.

1 Estimates of *p* values for linear trends were based on linear scores derived from the medians of the tertiles of fermented food intake among all participants.

2 Each point corresponds to a 10-unit increase in fermented food intake.

### 3.3 Age-stratified analysis

**Figure 2:**
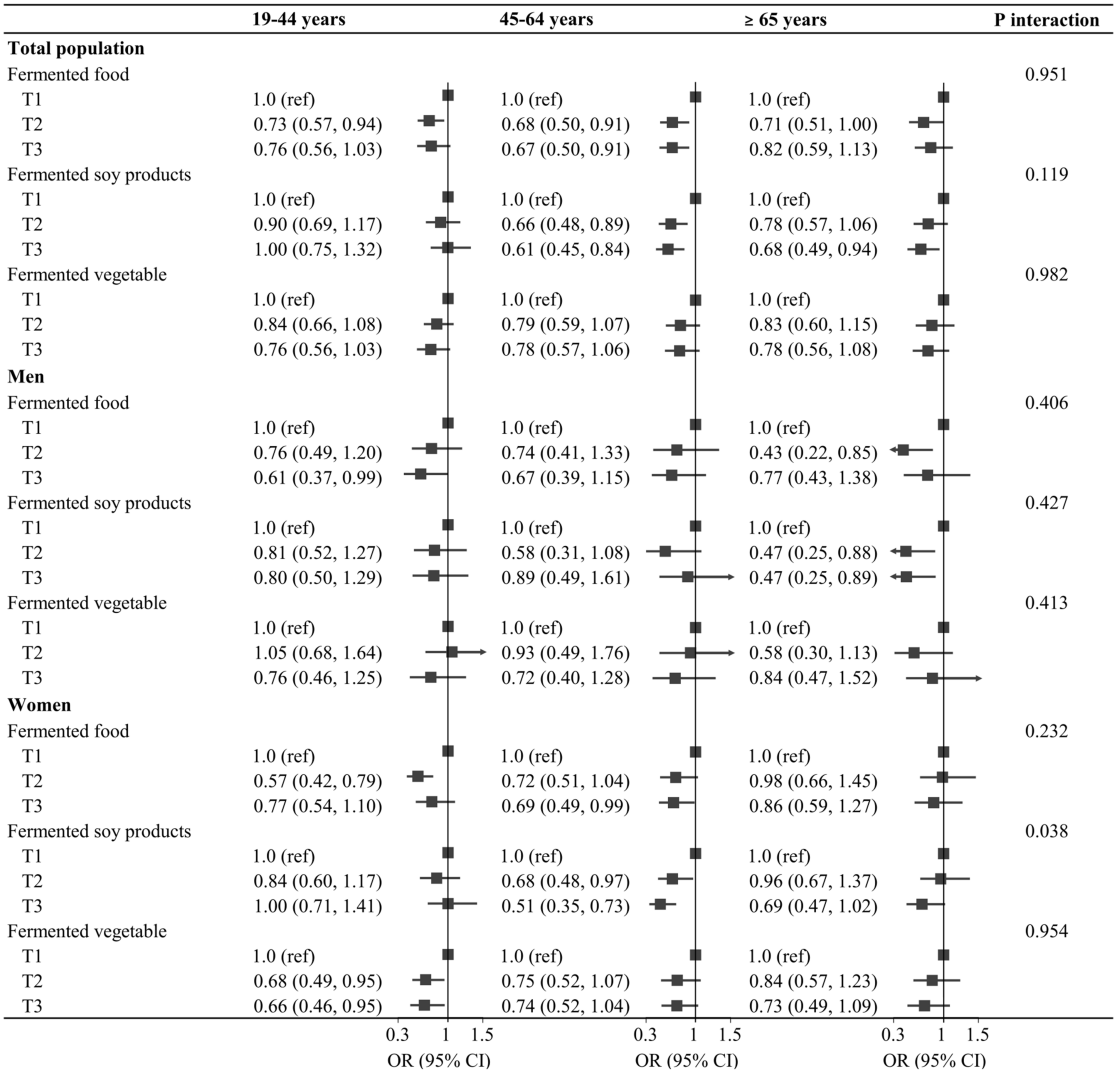
Age-stratified associations between fermented food intake and prevalence of depressive symptoms in the study population.

There was a significant age-related interaction in the association between the intake of fermented soy products and prevalence of depressive symptoms in women, with low risk in the higher tertiles among women aged 45–64 years (Figure 2). There was no significant age-related interaction between fermented food intake and suicidal ideation in the total population, men and women (Supplementary Figure 1).

## 4 Discussion

The present study demonstrated that the intake of fermented foods, soy products, and vegetables was inversely associated with the prevalence of moderate depressive symptoms in the total population of Korean adults. Further, the prevalence of depressive symptoms was inversely associated with the intake of fermented soy products and vegetables in women and fermented dairy products in men. Previous studies consistently reported that the prevalence of depression was inversely associated with the intake of fermented foods including fermented dairy products and *kimchi* in Korean adults (13), and fermented foods including fermented dairy products, *kimchi*, and *natto* in American adults (14). Depression scores are also inversely associated with intake of fermented foods including fermented dairy products, vegetables, and beverages in Polish students (15). In addition, the estimated intake of live microbes, categorized by the microbial content in each food, was inversely associated with the prevalence of depression among American adults from NHANES 2005–2018 (10, 11) and 2007–2016 (12). A systematic review of observational studies reported that dysbiosis of gut microbes was commonly observed in patients with depression, characterized by lower levels of butyrate-producing bacteria, such as *Faecalibacterium* and *Coprococcus,* and higher levels of pro-inflammatory bacteria, such as *Eggerthella* and *Streptococcus,* than those in healthy controls (7). A recent meta-analysis of randomized controlled trials also found that supplementation with probiotics, prebiotics, or synbiotics significantly alleviated depressive symptoms in Asian and Western population (9). Our previous study demonstrated that probiotics and postbiotics exert antidepressant-like effects through the brain-gut axis by enhancing microbiome abundance and short-chain fatty acids in the gut, and modulating the serotonergic pathway and hypothalamic–pituitary–adrenal axis in depressed rats induced by chronic mild stress (5).

There has been one previous study reporting an inverse association between fermented soy product intake and prevalence of depression in Japanese women (16). Furthermore, intake of soy products was inversely associated with the prevalence and incidence of depression in Chinese adults, including men and women (31–33). Previous studies have consistently shown a beneficial association between soy product intake and depression scores (34), and prevalence of depression (16) in Japanese women. The intake of soy isoflavones was inversely associated with the prevalence of depression in Japanese women (16), but not in men (35), supporting the sex-specific effect of soy. Supplementation of genistein, an isoflavone compound, altered gut microbiota composition, decreased intestinal inflammatory cell infiltration, and attenuated hypothalamic–pituitary–adrenal axis hyperactivity, ultimately alleviating depression-like behavior in rats with chronic unpredictable mild stress (36). Additionally, isoflavones metabolites exert estrogen-like effects by binding to human estrogen receptors *α* and *β* (37), and activation of estrogen receptors β demonstrated antidepressant-like effects in female, but not male rats (38). The primary form of soy isoflavones, glycosides, is converted into aglycones, such as genistein, during fermentation (39), which exhibit stronger estrogen-receptor binding than glycoside forms, suggesting a greater effect of fermented soy than that of soy itself (37).

In the present study, there was a significant age-related interaction between the intake of fermented soy products and the prevalence of depressive symptoms in women, showing an inverse association among those aged 45–64 years old. Similarly, a meta-analysis of randomized controlled trials reported that soy isoflavone supplementation alleviated depressive symptoms in perimenopausal women with mean ages ranging from 47 to 60 years (40). Prevalence of depression was higher in perimenopausal women than premenopausal women, which indicated that fluctuations of estrogen levels increased vulnerability to depression (41). During the perimenopausal period brain monoamine oxidase A (MAO-A), an enzyme that degrades serotonin, was higher than that during the premenopausal and postmenopausal periods, suggesting a higher risk of depression in the perimenopausal period, partly due to MAO-A (42). Estrogen significantly reduced brain MAO-A activity in ovariectomized rats (43) and further treatment with genistein decreased the enzymatic activity of recombinant human MAO-A obtained from transfected cells (44).

The intake of fermented vegetables accounted for approximately 75% of all fermented foods and was significantly associated with a lower prevalence of depressive symptoms in the present study. In fact, *kimchi* is a probiotic food rich in *Lactobacillus* and *Leuconostoc* species (45). Although there have been no previous studies on the intake of fermented vegetables, the intake of vegetables was inversely associated with the risk of depression in all adults (46) and women (47–50), but not in men (47, 50). Consistently, a meta-analysis of epidemiological studies found that dietary fiber, rich in vegetables, was inversely associated with the risk of depression in the total population and in women, but not in men (51). Supplementation with dietary fiber, a known prebiotic, increased the beneficial gut microbiota, enhanced fecal short-chain fatty acids, and restored brain serotonin in female mice with depression induced by a high-fat diet (52). Additionally, Wang et al. (53) showed that oral administration of a human fecal suspension enriched with beneficial bacteria by vegetable- and prebiotic-supplemented diets increased butyrate- and acetate-producing bacteria in female germ-free mice, but not in males. Santos-Marcos et al. (54) reported that short-chain fatty acid–producing genera such as *Prevotella*, *Roseburia*, and *Ruminococcus* were more abundant in Spanish women than men, suggesting the greater effect of vegetables on depression among women than men.

Consistent with the present study, the intake of fermented dairy products was inversely associated with both the prevalence and incidence of depression during a 24-year follow-up in Finnish men (17). The intake of yogurt or cheese was not significantly associated with the prevalence or incidence of depression in adults (20, 55, 56) or women (18–20). A meta-analysis of randomized controlled trials showed that the beneficial effects of probiotics, prebiotics, and synbiotics on depressive symptoms were greater in studies involving a higher proportion of men (9). Alpha diversity of the gut microbiome is negatively associated with depressive symptoms (57) and is lower in men than that in women in large cohort studies (58). Male mice had lower alpha diversity than castrated male mice due to the androgen-dependent effects (59), suggesting that sex differences on alpha diversity could be driven by hormonal influences. In addition, Asian cohorts including Korean showed that men consumed more meat, such as red meat and poultry than women (60), and intake of red meat was negatively associated with alpha diversity of the gut microbiome (61). Additionally, supplementation with probiotics restored the alpha diversity of the gut microbiota, which improved depression-like behaviors in male mice with depression induced by chronic stress (62).

Although there were no studies investigating the association between fermented seafood intake and the prevalence of depression, a meta-analysis of observational studies reported the inverse association between risk of depression and intake of fish or n-3 polyunsaturated fatty acids (PUFAs) (63). Supplementation of n-3 PUFAs modulated the gut microbiota composition, increased microbial short-chain fatty acid production, decreased gut permeability by reduction of endotoxin production, and reduced inflammatory cytokines, which improved depressive symptoms through serotonergic and dopaminergic pathways in depressed rats exposed to chronic mild stress (5, 6). However, the intake of n-3 PUFAs from fermented seafood may be insufficient to produce beneficial effects in depressive symptoms. The primary type of fermented seafood is *jeotgal*, which is used as a seasoning and has a high-salt content (64). The frequency of adding salt to food was positively associated with the incidence of depression in the UK Biobank Study (65).

The present study showed that the prevalence of suicidal ideation was inversely associated with the intake of fermented foods, soy products, and vegetables in the total population. Additionally, the intake of fermented soy products was inversely associated with the prevalence of suicidal ideation in women. Although there have been no studies regarding fermented food intake and suicidal ideation, dietary live microbes, estimated from the expected microbial content of foods, were inversely associated with the prevalence of suicidal ideation in American adults from the NHANES (10). Additionally, the gut microbiota related to inflammation, adrenal steroid hormones, and serotonin metabolism were higher in participants with suicidal ideation or completion than in those without suicidal ideation or completion (22–24). The prevalence of suicidal ideation was inversely associated with vegetable intake in Korean adults (66) and with fish consumption in Finnish adults (67). Moreover, the risk of death from suicide was inversely associated with dietary patterns that were high in vegetables, fruits, soy products, and fish among Japanese adults during a mean follow-up of 8.6 years (68).

To the best of our knowledge, this is the first study to investigate the association of depressive symptoms and suicidal ideation with the intake of fermented foods and their types using nationally representative data. However, this study had some limitations that should be considered when interpreting the results. First, the rationale for the study was based on the assumption that fermented food consumption alters the gut microbiota. However, the gut microbiome and metabolome were not measured. Second, dietary intake was assessed using a single 24-h recall method, which could have recall bias and might not represent the participants’ usual diet. Third, owing to its cross-sectional design, the study was unable to establish a causal relationship between fermented food intake and the prevalence of depressive symptoms and suicidal ideation. Fourth, adjustments were made for various potential confounders, such as age, sex, BMI, education, household income, smoking status, physical activity, and unemployment. However, the possibility of residual confounders, including the medical history of chronic diseases such as cardiovascular disease, remains. Lastly, the exclusion of participants with missing baseline characteristics could introduce a potential source of bias.

## 5 Conclusion

The intake of fermented food was inversely associated with the prevalence of moderate depressive symptoms and suicidal ideation, with sex-specific differences according to the fermented food type, suggesting that fermented food intake could be beneficial in reducing the risk of depressive symptoms and suicidal ideation in Korean adults. Further studies are needed to confirm the effects of fermented food intake on depression through clinical trials and the incidence of depression through long-term follow-up cohort studies.

## Data Availability

Publicly available datasets were analyzed in this study. This data can be found at: https://knhanes.kdca.go.kr/knhanes/main.do.
