# Supplementary material for: Association of fermented food intake with the prevalence of depressive symptoms and suicidal ideation in men and women stratified by age: the Korea National Health and Nutrition Examination Survey 2014–2022
Source: Front Nutr. 2026 Jan 30;13:1707954. doi: 10.3389/fnut.2026.1707954 (PMC12900688; doi:10.3389/fnut.2026.1707954)
Supplement: Supplementary file 1 [file Table_1.docx]

Supplementary Material

Supplementary Table 1. Fermented food intake of participants with and without depressive symptoms

| Intake (g/day) | Total population | | *p*  value | Men | | *p* value | Women | | *p* value |
| --- | --- | --- | --- | --- | --- | --- | --- | --- | --- |
|  | Without  depressive symptoms (n = 20,072) | With  depressive symptoms (n = 1,124) |  | Without  depressive symptoms (n = 8,429) | With  depressive symptoms (n = 318) |  | Without  depressive symptoms (n = 11,643) | With  depressive symptoms (n = 806) |  |
| Fermented food | 139.21 ± 1.60 | 128.51 ± 4.31 | 0.014 | 158.71 ± 2.22 | 147.41 ± 8.49 | 0.197 | 120.35 ± 2.73 | 109.92 ± 5.20 | 0.024 |
| Fermented soy products | 18.08 ± 0.28 | 16.30 ± 0.71 | 0.013 | 19.79 ± 0.37 | 17.86 ± 1.40 | 0.180 | 15.94 ± 0.47 | 14.11 ± 0.88 | 0.022 |
| Fermented vegetable | 106.64 ± 1.46 | 99.81 ± 3.84 | 0.080 | 126.20 ± 2.04 | 122.37 ± 8.27 | 0.654 | 86.15 ± 2.28 | 77.94 ± 4.07 | 0.026 |
| Fermented dairy products | 13.04 ± 0.60 | 10.94 ± 1.99 | 0.294 | 10.98 ± 0.80 | 5.05 ± 1.76 | 0.002 | 17.19 ± 1.40 | 17.01 ± 3.43 | 0.949 |
| Fermented seafood | 1.46 ± 0.07 | 1.46 ± 0.18 | 0.981 | 1.74 ± 0.10 | 2.12 ± 0.44 | 0.397 | 1.07 ± 0.09 | 0.86 ± 0.14 | 0.156 |

Data are presented as weighted mean ± standard error of the mean. *p*-values were determined using analysis of covariance (ANCOVA) for continuous variables after adjusting for age, sex, BMI, education, household income, smoking status, physical activity, and unemployment.


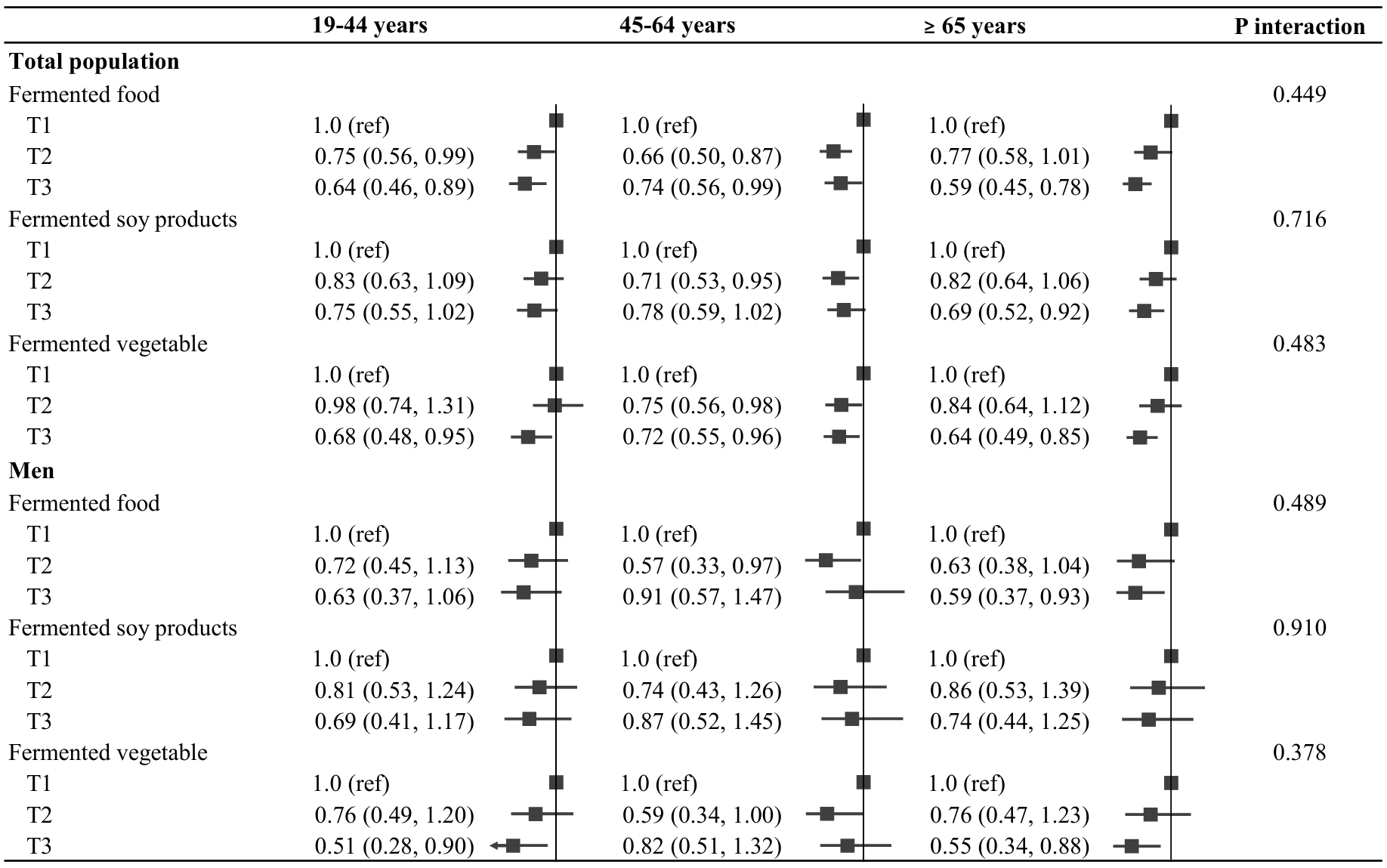


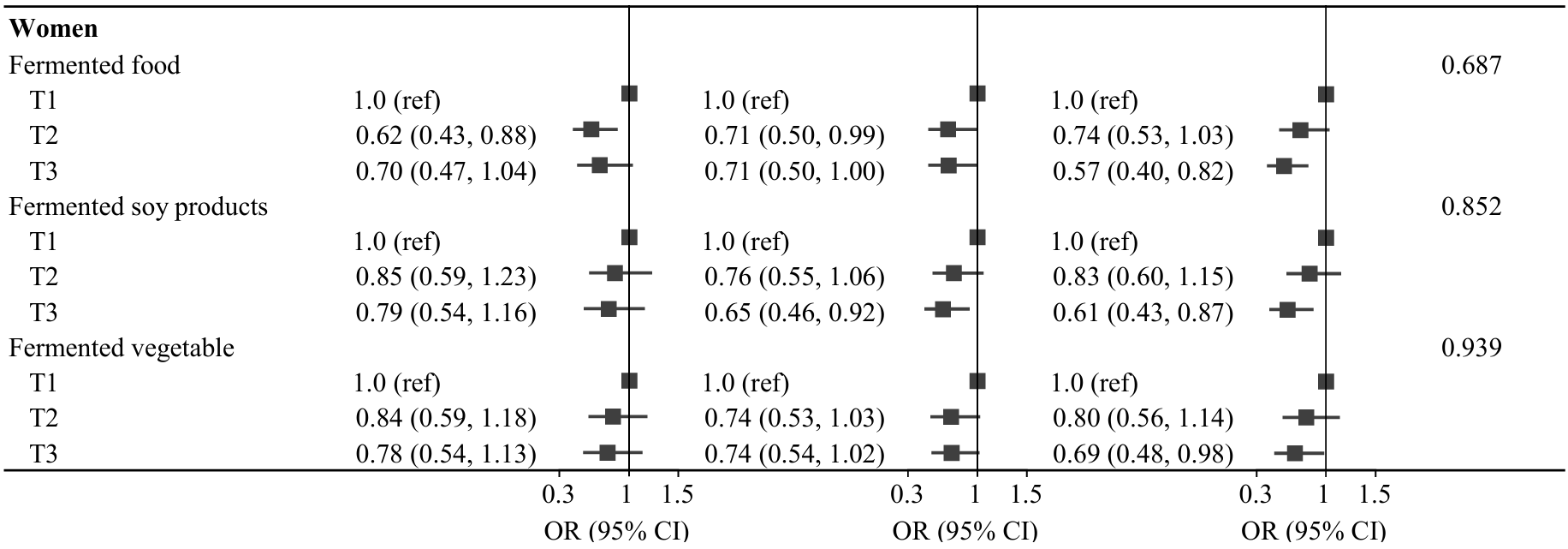


Supplementary Figure 1. Age-stratified associations between fermented food intake and prevalence of suicidal ideation in the study population
